# Supplementary material for: Possibility for strong northern hemisphere high-latitude cooling under negative emissions
Source: Nat Commun. 2022 Mar 1;13:1095. doi: 10.1038/s41467-022-28573-5 (PMC8888562; doi:10.1038/s41467-022-28573-5)
Supplement: Supplementary file 1 — Supplementary Information [file 41467_2022_28573_MOESM1_ESM.pdf]

Supplementary Material

Possibility for strong northern hemisphere high-latitude cooling under negative emissions

Jörg Schwinger[1,*], Ali Asaadi[1], Nadine Goris[1], Hanna Lee[1,2]

[1]NORCE Climate, Bjerknes Centre for Climate Research, Bergen, Norway

[2]Norwegian University of Science and Technology, Trondheim, Norway

[*]Corresponding author, jorg.schwinger@norceresearch.no

Supplementary Figures:

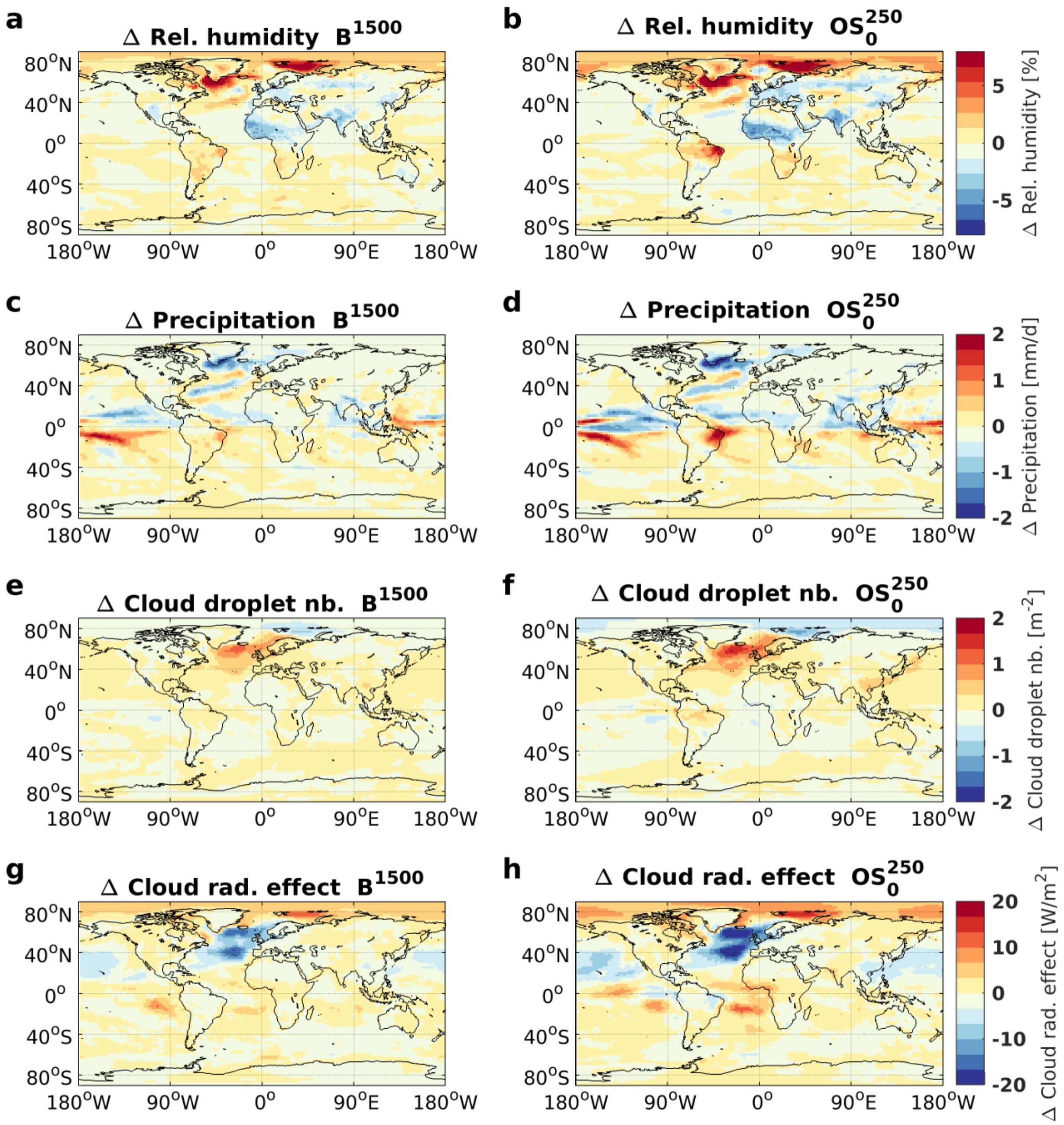

Supplementary figure S1: Difference between a state with reduced AMOC around the SAT minimum (years 180-190) and a state with recovered AMOC and SAT (years 280-289) for the reference simulation B^{1500} (left column) and the OS_0^{250} overshoot (right column). Shown is the mean of three ensemble members for (a,b) surface relative humidity, (c,d) precipitation, (e,f) vertically integrated cloud droplet number, and (g,h) cloud radiative effect.

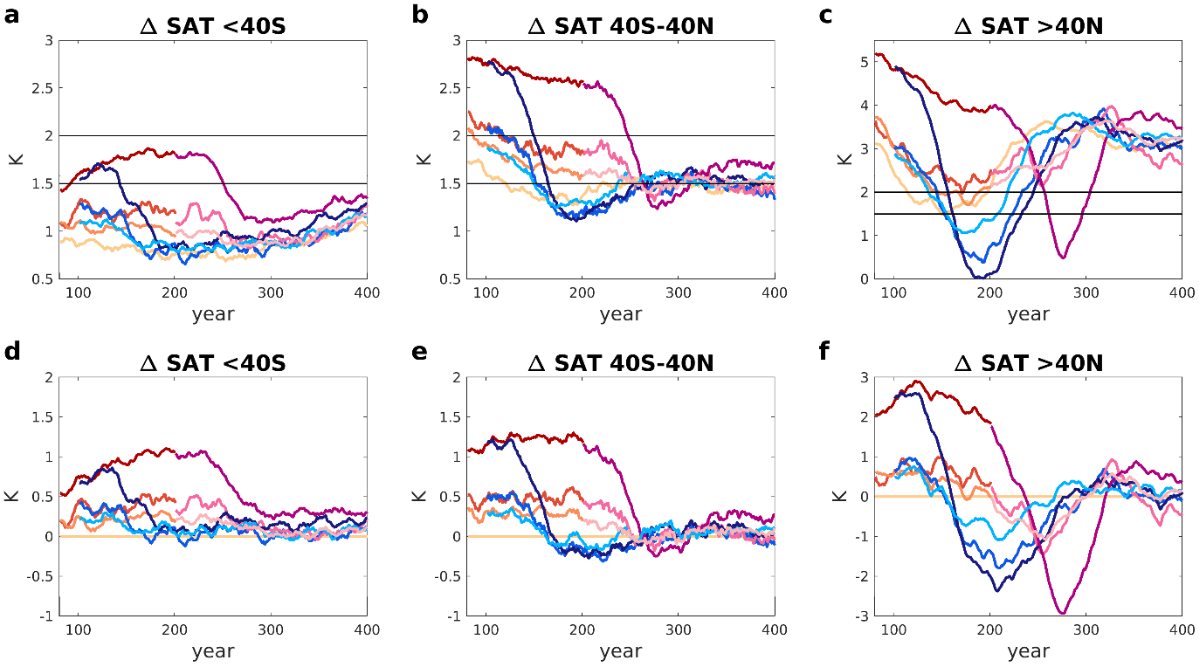

Supplementary figure S2: Change in average SAT relative to pre-industrial conditions smoothed by an 11-year running mean (a) south of 40°S, (b) between 40°S and 40°N, (c) north of 40°N. Panels d-f display the average SAT relative to the reference simulation B^{1500} for the same regions as panels a-c. For clarity, only the ensemble means of the OS (blue and magenta lines) and B-simulations (yellow to red lines) are shown (see Fig. 1 for color codes).

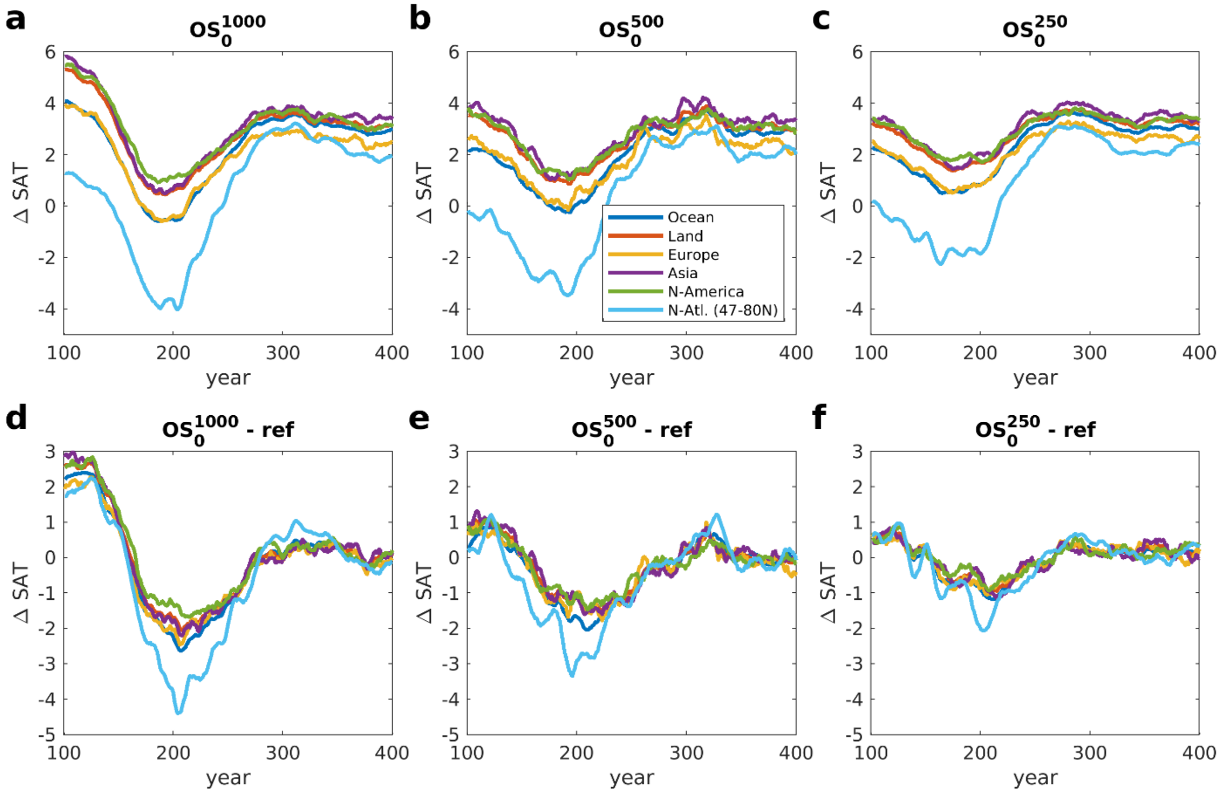

Supplementary figure S3: Difference in (a-c) surface air temperature north of 40°N relative to the pre-industrial control simulation for the three short overshoots as indicated in the panel title, and (d-f) same as panels a-c but relative to the reference simulation B^{1500} (without overshoot). Shown are temperature differences over the ocean (blue lines), the North Atlantic between 47° and 80°N (light blue lines), over land (red lines), Europe (yellow lines), Asia (purple lines), and North America (green lines).

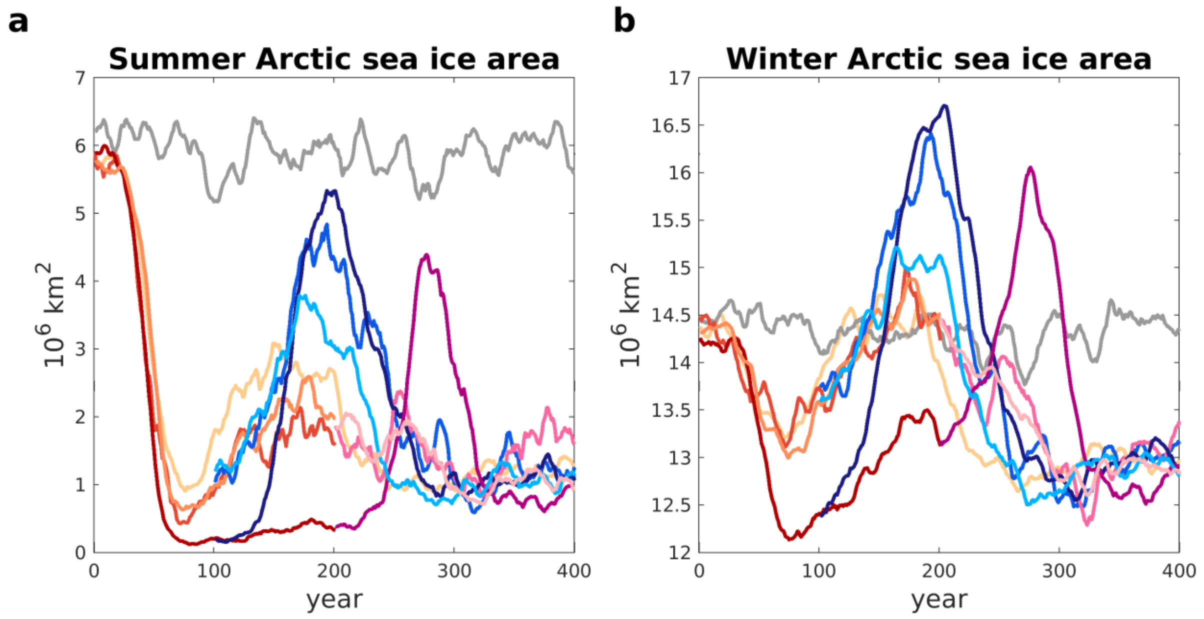

Supplementary figure S4: (a) Summer (September) and (b) winter (March) sea-ice extent smoothed by an 11-year running mean. For clarity, only the ensemble means of the OS- (blue and magenta lines) and B-simulations (yellow to red lines) are shown (see Fig. 1 for color codes).

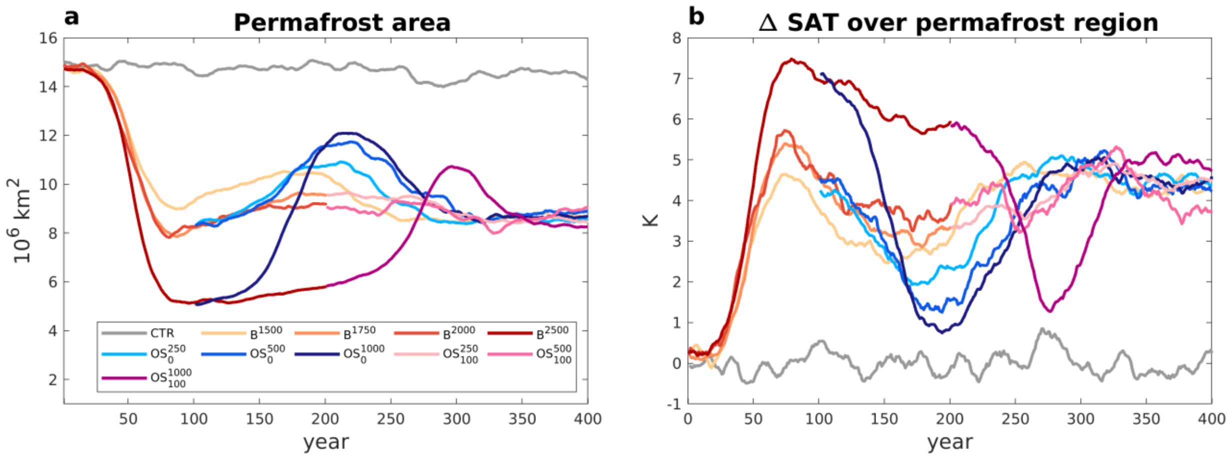

Supplementary figure S5: Changes in (a) permafrost area north of 45°N and (b) average SAT over the region with permafrost soil in the pre-industrial control simulation. An 11-year running mean has been applied to the data.

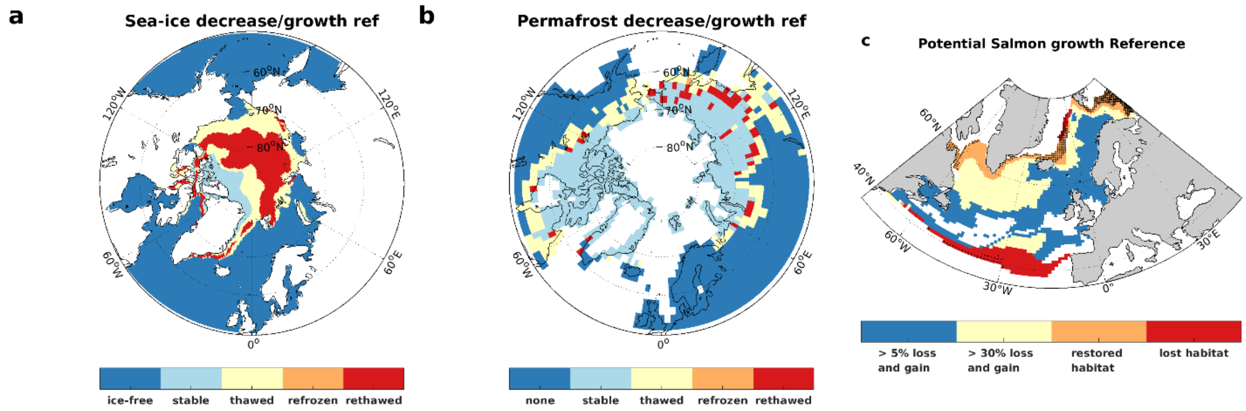

Supplementary figure S6: As figure 3 but for the reference simulation B^{1500}.

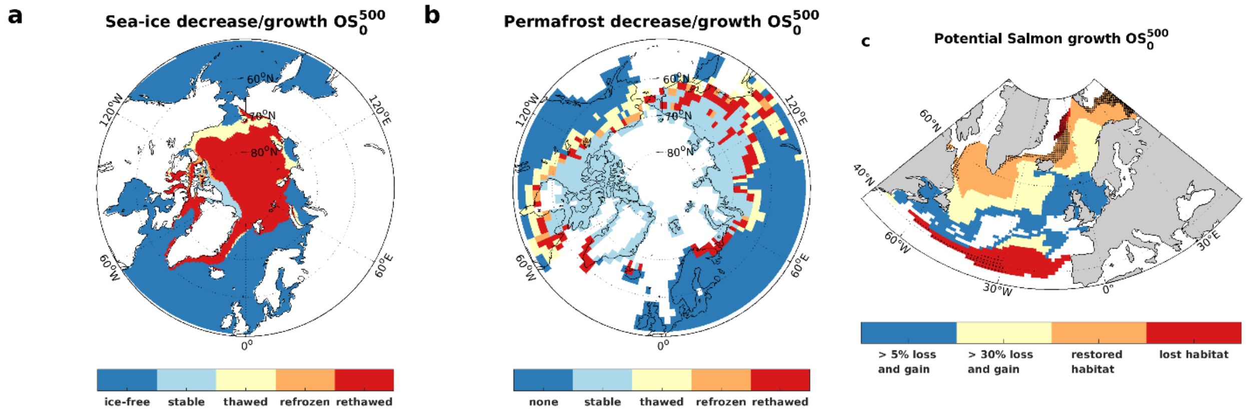

Supplementary figure S7: As figure 3 but for the medium overshoot OS_0^{500}.

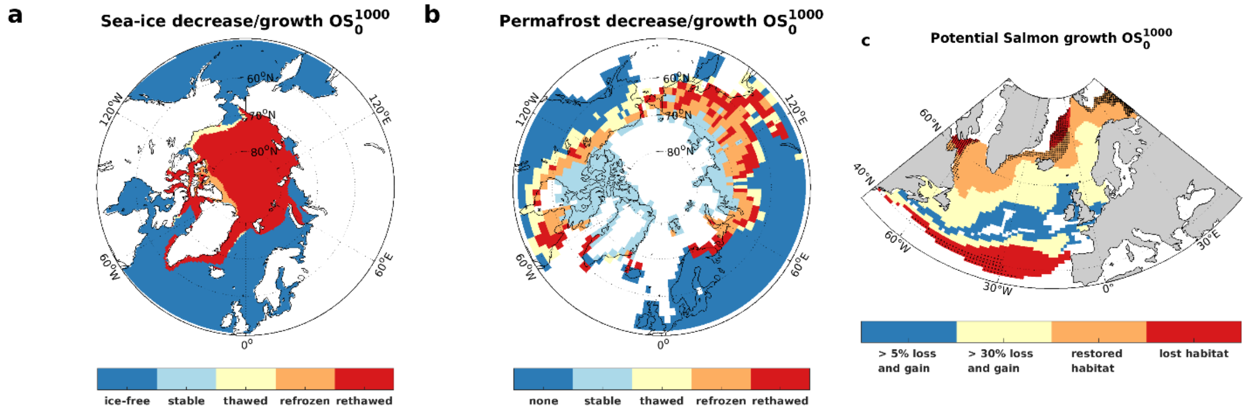

Supplementary figure S8: As figure 3 but for the high overshoot OS_0^{1000}.

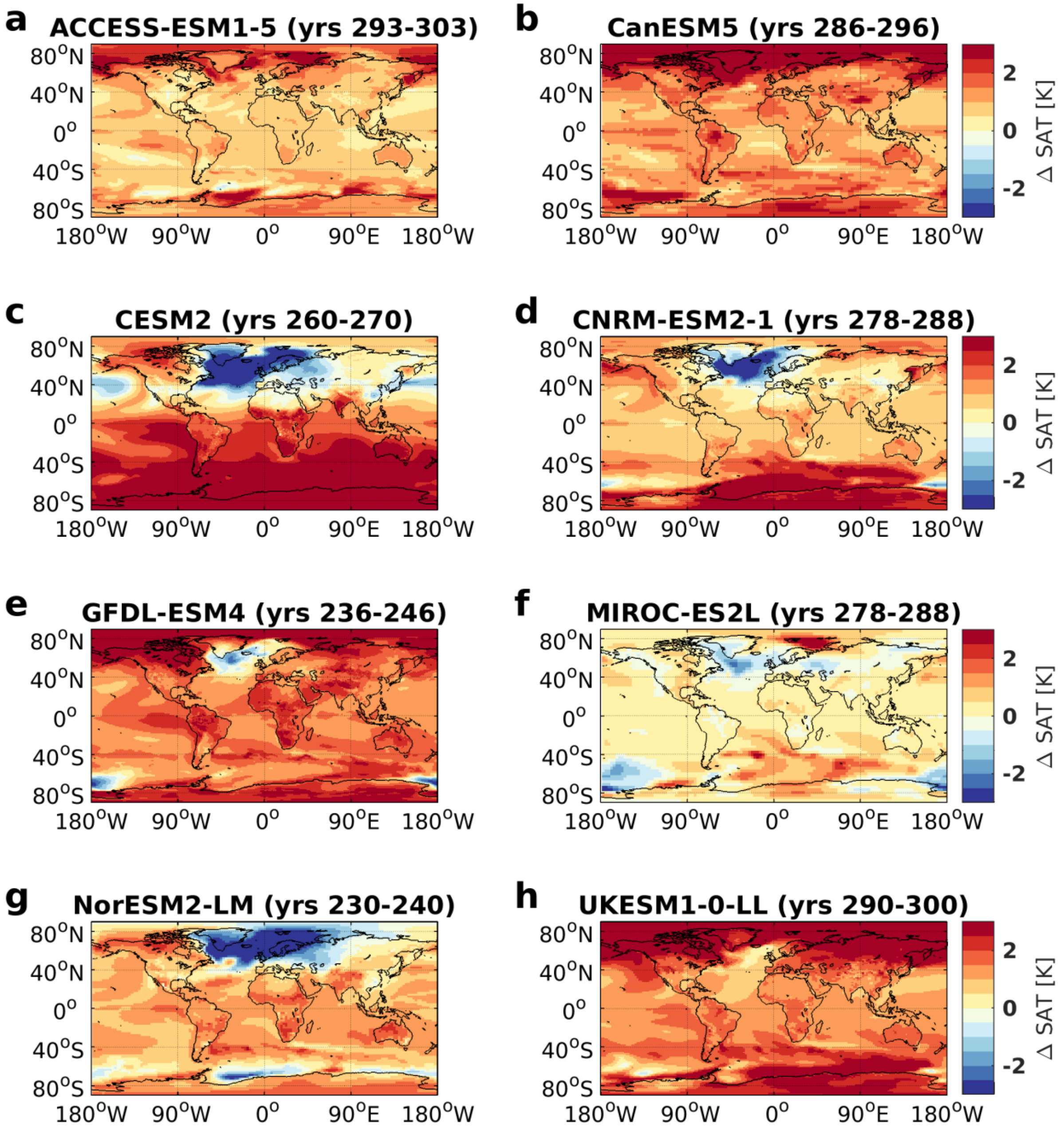

Supplementary figure S9: Results from the multi-model intercomparison CDRMIP[17]: Difference between SAT in the 1pctCO2-cdr simulation and the pre-industrial control simulation for the years (indicated in the panel title) around the temperature minimum in the North Atlantic region (compare Fig. 4a-c). Model output for the 1pctCO2-cdr simulation is available (at the time of writing) for NorESM2-LM and seven other ESMs[45–51] as indicated in the panel title.
